# Supplementary material for: Reconciling Mining with the Conservation of Cave Biodiversity: A Quantitative Baseline to Help Establish Conservation Priorities
Source: PLoS One. 2016 Dec 20;11(12):e0168348. doi: 10.1371/journal.pone.0168348 (PMC5173368; doi:10.1371/journal.pone.0168348)
Supplement: S1 Dataset — (ZIP) [file pone.0168348.s002.zip › Taxa/Serra Norte/SN_2013/N5S/Lista N5S-14.pdf]

CAVIDADE N5S-0014

Classe	Ordem	Fam/Outros	Gên/Outros	Espécie	S-U	U-S	Zon.
Annelida	Oligochaeta			jovem	1		
Arachnida	Acari	Eupodidae		sp.1	X		P/A
Arachnida	Acari	Laelapidae		sp.3	X		P
Arachnida	Acari	Laelapidae		sp.73	X		E
Arachnida	Acari	Rhagidiidae		sp.20	X		E/P
Arachnida	Acari	Trombiculidae		sp.39	X		A
Arachnida	Acari	Trombidiformes		sp.49	X		P/A
Arachnida	Amblypygi	Charinidae	*Charinus*	sp.1	4		P/A
Arachnida	Amblypygi	Phrynidae	*Heterophrynus*	*longicornis*		X	
Arachnida	Araneae	Araneidae	*Alpaida*	sp.1		X	
Arachnida	Araneae	Araneidae	*Micrathena*	*plana*	X		
Arachnida	Araneae	Barychelidae		jovem	1		E
Arachnida	Araneae	Caponiidae		sp.1	X	X	P/A
Arachnida	Araneae	Corinnidae		jovem	1		P
Arachnida	Araneae	Ctenidae	*Ctenus*	sp.2	4		P
Arachnida	Araneae	Drymusidae		jovem	X		P
Arachnida	Araneae	Mysmenidae	*Microdipoena*	sp.1	X		E/P
Arachnida	Araneae	Ochyroceratidae	*Ochyrocera*	sp.3	X		P
Arachnida	Araneae	Ochyroceratidae	*Speocera*	sp.1	X		P
Arachnida	Araneae	Pholcidae	*Leptopholcus*	sp.1	X		E
Arachnida	Araneae	Pholcidae	*Mesabolivar*	aff. *cambridgei*	X		P
Arachnida	Araneae	Pholcidae	*Mesabolivar*	sp.1	X		P
Arachnida	Araneae	Prodidomidae	*Lygromma*	sp.2	X		P
Arachnida	Araneae	Salticidae		sp.7	X		E/P
Arachnida	Araneae	Salticidae		sp.10	X		P
Arachnida	Araneae	Scytodidae	*Scytodes*	sp.		X	
Arachnida	Araneae	Segestriidae		jovem	X		P
Arachnida	Araneae	Theraphosidae		sp.		X	
Arachnida	Araneae	Theridiidae	*Theridion*	sp.1	X		E
Arachnida	Araneae	Theridiosomatidae	*Plato*	sp.1	X		P
Arachnida	Opiliones	Escadabiidae		sp.2	X		A
Arachnida	Opiliones	Sclerosomatidae	*Prionostema*	sp.1	2		P
Arachnida	Pseudoscorpiones	Bochicidae		jovem	X		P
Arachnida	Pseudoscorpiones	Chernetidae	*Spelaeochernes*	sp.1	X	X	E/P/A
Chilopoda	Scolopendromorpha	Cryptopidae	*Cryptops*	sp.		X	
Chilopoda	Scolopendromorpha	Scolopocryptopidae	*Tidops*	sp.1	1		P
Chilopoda	Scutigeromorpha	Pselliodidae	*Sphendononema*	jovem	6		P/A
Diplopoda	Polydesmida	Pyrgodesmidae		sp.1	X	X	P
Entognatha	Collembola	Cyphoderidae		sp.1	X		P
Entognatha	Collembola	Isotomidae		sp.1	X		P
Entognatha	Collembola	Paronellidae		sp.1	X		E/P
Entognatha	Collembola	Sminthuroidea		sp.2	X		P
Entognatha	Diplura	Campodeidae		sp.1	X	X	E/P
Entognatha	Diplura	Japygidae		sp.3	X		E
Insecta	Blattodea			jovem	4		E/P
Insecta	Coleoptera	Curculionidae	Platypodinae	sp.1	X		P
Insecta	Coleoptera	Staphylinidae	Pselaphinae	sp.12	X		P
Insecta	Diptera	Chironomidae		sp.	X		E
Insecta	Diptera	Chloropidae		sp.	X		E

Insecta	Diptera	Culicidae	*Culex*	sp.	X		P
Insecta	Diptera	Psychodidae	Phlebotominae	sp.	X		E/P/A
Insecta	Heteroptera	Reduviidae	Emesinae	sp.1	1	X	P
Insecta	Heteroptera	Reduviidae	Reduviinae	sp.	4		E/P
Insecta	Homoptera	Cixiidae		jovem	X	X	E/P/A
Insecta	Hymenoptera	Formicidae	*Brachymyrmex*	sp.1	X		E/P
Insecta	Hymenoptera	Formicidae	*Brachymyrmex*	sp.2	X		P
Insecta	Hymenoptera	Formicidae	*Dolichoderus*	aff. *bispinosus*	X		E
Insecta	Hymenoptera	Formicidae	*Hypoponera*	sp.1	X		E
Insecta	Hymenoptera	Formicidae	*Hypoponera*	sp.2	X		E
Insecta	Hymenoptera	Formicidae	Myrmicinae	sp.1	X		P
Insecta	Hymenoptera	Formicidae	*Pachycondyla*	sp.1	X		P
Insecta	Hymenoptera	Formicidae	*Paratrechina*	sp.1	X		P
Insecta	Hymenoptera	Formicidae	*Paratrechina*	sp.2	X		P
Insecta	Hymenoptera	Formicidae	*Pheidole*	sp.1	X		P
Insecta	Hymenoptera	Formicidae	*Pheidole*	sp.3	X		P
Insecta	Hymenoptera	Formicidae	*Solenopsis*	sp.1	X		P
Insecta	Hymenoptera	Formicidae	*Trachymyrmex*	sp.1	X		P
Insecta	Hymenoptera	Ichneumonidae		sp.2	X		P
Insecta	Isoptera	Termitidae		sp.	X	X	
Insecta	Lepidoptera	Gelechioidea		sp.	X		E/P
Insecta	Lepidoptera	Noctuoidea		sp.1	1		
Insecta	Lepidoptera	Tineoidea		sp.	X		P
Insecta	Orthoptera	Phalangopsidae	*Paraclodes*	jovem	2	X	
Insecta	Orthoptera	Phalangopsidae	*Phalangopsis*	sp.	30	X	
Insecta	Psocoptera	Epipsocidae	*Epipsocus*	sp.2	X		E/P
Insecta	Thysanura	Ateluridae		sp.1	X		P
Insecta	Thysanura	Nicoletiidae		sp.1	X		E/P
Gastropoda	Pulmonata	Subulinidae	*Leptinaria*	sp.		X	
Gastropoda	Pulmonata	Systrophiidae	*Happia*	sp.	X	X	P
Malacostraca	Isopoda	Philosciidae		sp.		X	
Malacostraca	Isopoda	Scleropactidae	*Circoniscus*	*buckupi*	X		P
Onychophora	Onychophora	Peripatidae	*Peripatus*	sp.1	1		E
Symphyla		Scutigerellidae		sp.	X		E
Amphibia	Anura	Bufonidae	*Rhinella*	sp.	4		E/P
Mammalia	Chiroptera	Emballonuridae	*Peropteryx*	*kappleri*	5		E/P
Mammalia	Chiroptera	Furipteridae	*Furipterus*	*horrens*	15		P/A
Mammalia	Chiroptera	Phyllostomidae	*Trachops*	*cirrhosus*	1		P/A
